# Complications associated with surgical repair of syndromic scoliosis

**DOI:** 10.1186/s13013-015-0035-x

**Published:** 2015-04-23

**Authors:** Benjamin J Levy, Jacob F Schulz, Eric D Fornari, Adam L Wollowick

**Affiliations:** Montefiore Medical Center and Albert Einstein College of Medicine, 1250 Waters Place, 11th Floor, Bronx, NY 10461 USA; Orthopaedic Surgery, Albert Einstein College of Medicine, Bronx, USA

**Keywords:** Syndromic, Scoliosis, Complications, Surgical, Down, Marfan, Rett, Nuerofibromatosis

## Abstract

**Background:**

There are a number of syndromes that have historically been associated with scoliosis e.g.: Marfan, Down, and Neurofibromatosis. These syndromes have been grouped together as one etiology of scoliosis, known as syndromic scoliosis. While multiple studies indicate that these patients are at high risk for perioperative complications, there is a paucity of literature regarding the collective complication rates and surgical needs of this population.

**Methods:**

PubMed and Embase databases were searched for literature encompassing the surgical complications associated with the surgical management of patients undergoing correction of scoliosis in the syndromic scoliosis population. Following exclusion criteria, 24 articles were analyzed for data regarding these complications.

**Results:**

The collective complication rates and findings of these articles were categorized based on specific syndrome. The rates and types of complications for each syndrome and the special needs of patients with each syndrome are discussed. Several complication trends of note were observed, including but not limited to the universally nearly high rate of wound infections (>5% in each group), high rate of pulmonary complications in patients with Rett syndrome (29.2%), high rate (>10%) of dural tears in Marfan and Ehlers-Danlos syndrome patients, high rate (>20%) of implant failure in Down and Prader-Willi syndrome patients, and high rate (>25%) of pseudarthrosis in Down and Ehlers-Danlos patients.

**Conclusions:**

Though these syndromes have been classically grouped together under the umbrella term “syndromic,” there may be specific needs for patients with each of these ailments. Given the high rate of complications, further research is necessary to understand the unique needs for each of these patient groups in the preoperative, intraoperative, and postoperative settings.

## Introduction

Scoliosis in the pediatric population is traditionally classified as idiopathic, congenital, neuromuscular, or syndromic in etiology. Syndromic scoliosis is generally recognized as scoliosis that is associated with a systemic disease. Diseases associated with scoliosis include, but are not limited to, Down syndrome, Marfan syndrome, neurofibromatosis, Rett syndrome, achondroplasia, Ehlers-Danlos syndrome, Prader-Willi syndrome, Friedrich’s ataxia, and Osteogenesis Imperfecta. In these conditions, scoliosis occurs at a rate significantly greater than that of the population at large. Despite the usage of the umbrella term “syndromic scoliosis,” there is a paucity of literature that discusses these conditions as one entity. Moreover, little has been reported in the literature about the collective complication rates, or potential for a unique complication profile, associated with surgical management of syndromic scoliosis.

The surgical management of scoliosis is associated with significant complications including blood loss, infection, and neurologic compromise. It has previously been demonstrated that patients with non-idiopathic scoliosis, especially neuromuscular, have higher rates of infection, blood loss during surgery, and subsequent need for transfusion, than idiopathic scoliosis patients [1]. It should be noted that non-idiopathic patients also have significantly higher rates of medical comorbidities compared to idiopathic patients [2]. The syndromic population, with their resultant spinal deformities and medical comorbidities, are likely to have a significant complication profile as well. Understanding more clearly the rates and types of complications seen in the syndromic scoliosis population is an important step in allowing for us to improve the care of these individuals.

The aim of this article is to collect and group data regarding the complications associated with the repair of syndromic scoliosis. With this data, future research can examine the special needs of these patients in the peri-operative period to improve their care.

For the purposes of this review, all syndromic cases should be viewed as being in the same category, thus delineating them from idiopathic, congenital or neuromuscular scoliosis. The surgical repair of scoliosis in the syndromic group has been associated with a high degree of intraoperative and postoperative complications based on anecdotal experience. There remains a gap in the literature regarding the rates and specifics of surgical complications.

## Methods

In order to fully encompass as much literature as possible, two databases were investigated to capture articles for this literature review according to the methods of Sharma et al [3]. Pubmed and Embase were both searched for the articles that were collected. Advanced search techniques were performed in both cases. Retrospective and Prospective studies were included, case reports that had fewer than 4 patients were not included for the purposes of this review. Articles were limited to the years 1990-2013 in an effort to homogenize data as much as possible. Additional limits on the search criteria were limited to limiting the language of the articles located to “English,” and relevance to the purpose of this study, scoliosis-surgery-related complications.

In total, this yielded 520 articles (not accounting for overlap) from Pubmed and 70 articles from Embase. Following exclusion criteria as stated above, 24 articles were analyzed, comprising 482 patients.

Articles were analyzed and data were extracted from articles in the areas of most common surgical complications reported. Complications were quantified in the areas of dural tear/leak, wound infections (superficial or deep), “excessive bleeding”, respiratory issues (pneumothorax, pneumonia etc), neurological compromise (paralysis, etc), re-operation (cause determined by individual surgeons), hardware failure (rod fracture, etc), and pseudarthroses. Average blood loss, age at time of (first) surgery, approach, and instrument type were collected for each article when available.

For each syndrome reviewed, summary tables have been compiled. These tables document collective rates of dural tears, wound infections, respiratory issues, neurological compromises, hardware failure, pseudarthrosis, and average blood loss in mL because these were the most consistently reported complications. Because not every study reported on every complication, complication rates are reported in both percentage and absolute values to indicate the variable denominator for each complication. As an example, 6 studies were included on Marfan syndrome comprising 139 surgical patients. Only 4 of these studies reported on dural tears and therefore the reference population for dural tears is 93 instead of 139.

## Results

(Table 1).

**Table 1:** **Syndromic scoliosis vs. AIS surgery complications**

	**Dural tear**	**Wound infection**	**Respiratory issues**	**Neuro compromise**	**Hardware failure**	**Pseudarthrosis**	**Avg blood loss mL (#)**
Marfan Pts (# patients reported/total)	11.8% (11/93)	6.5% (6/93)	5.7% (4/70)	2.2% (1/46)	17.2% (20/116)	6.2% (8/129)	1756 (106)
Down Pts (# patients reported/total)	Not Reported	14.3% (2/14)	14.3% (1/7)	Not Reported	35.7% (5/14)	28.6% (4/14)	1264 (7)
Rett Pts (# patients reported/total)	Not Reported	10.1% (9/89)	29.2% (26/89)	Not Reported	4.5% (3/66)	0% (0/16)	2106 (16)
NF Pts (# patients reported/total)	5.9% (3/51)	10.2% (9/88)	Not Reported	5.7% (5/88)	4.2% (4/96)	5.7% (5/88)	903 (51)
ED Pts (# patients reported/total)	16.7% (1/6)	Not Reported	20% (1/5)	0% (0/16)	22.7% (5/22)	36.3% (4/11)	1272 (22)
OI Pts (# patients reported/total)	Not Reported	5.0% (1/20)	Not Reported	5% (1/20)	12.8% (6/47)	5 % (1/20)	Not Reported
PW Pts (# patients reported/total)	Not Reported	22.7% (5/22)	6.3% (1/16)	22.7% (5/22)	22.7% (5/22)	4.5% (1/22)	1750 (6)
FA Pts (# patients reported/total)	Not Reported	6.3% (1/16)	6.3% (1/16)	0% (0/16)	6.3% (1/16)	Not Reported	1268 (16)
	Dural tear (Durotomy)	Wound infection (Deep and superficial)	Respiratory issues (Pulmonary) + (PE)	Neuro compromise (New neuro compromise) + (Peripheral nerve)	Hardware malfunction (Implant related)	Pseudarthrosis	Avg blood loss mL (#)
AIS Pts (# patients reported/total)	0.2% (22/11,227)	1.4% (156/11,227)	0.6% + 0.04% (63/11,227 + 5/11,227)	0.8% + 0.5% (86/11,227 + 53/11,227)	1.1% (120/11,227)	Not Reported	323-907-1277

### Marfan syndrome

**Figure 1:**
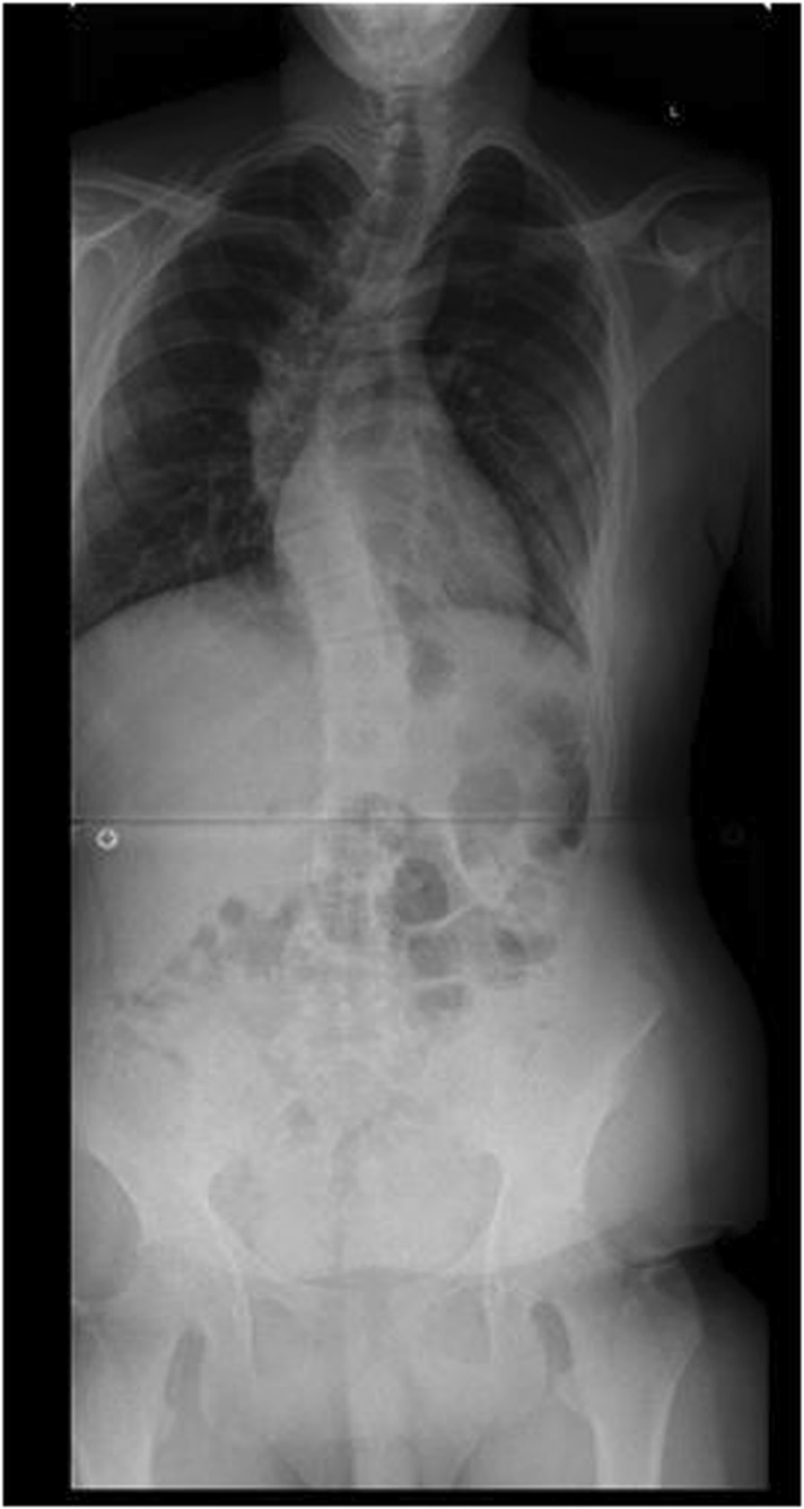
Pre-operative radiograph of 15 year-old male with Marfan syndrome.

**Figure 2:**
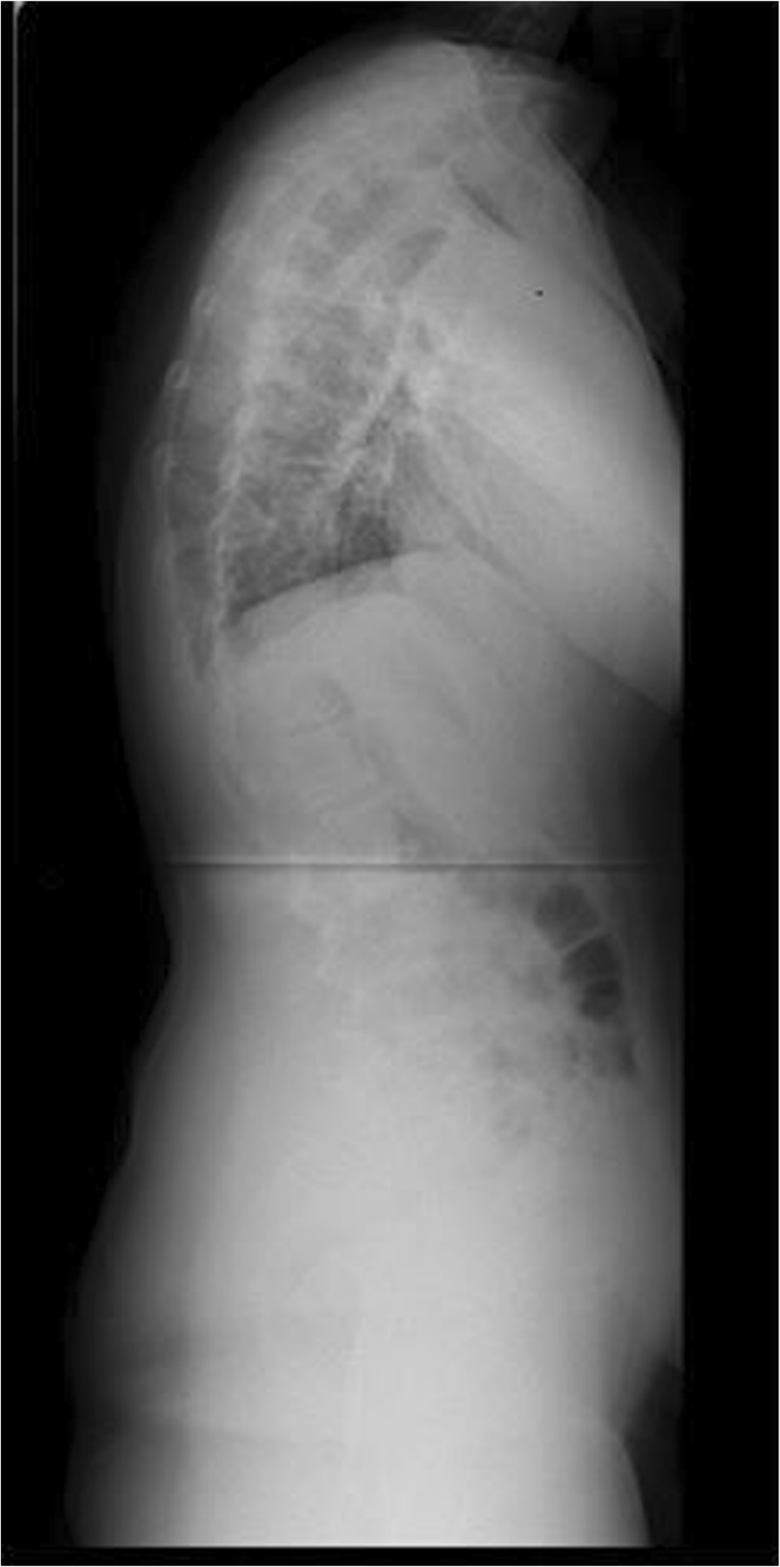
Pre-operative radiograph of 15 year-old male with Marfan syndrome.

**Figure 3:**
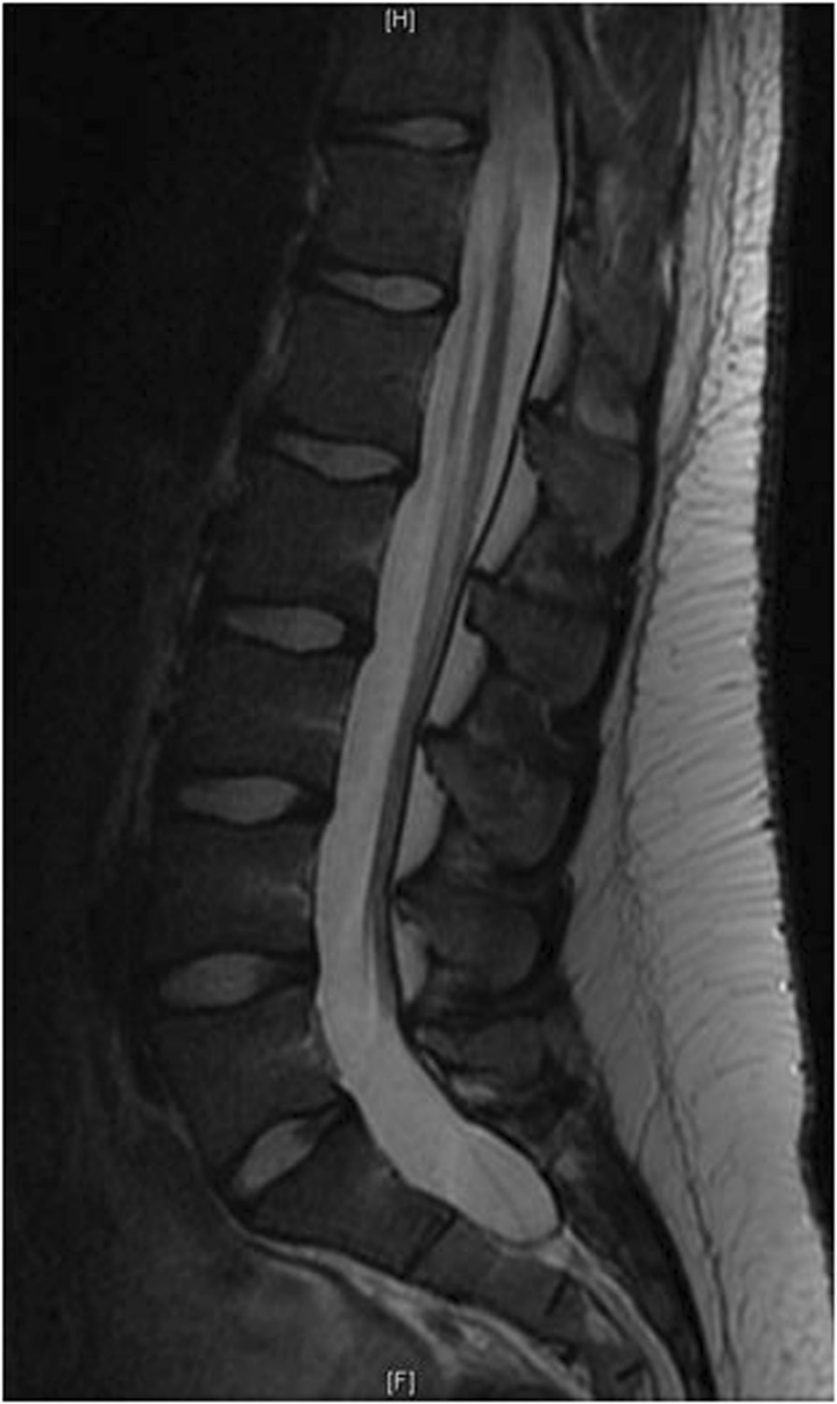
Pre-operative MRI of a representative patient with Marfan syndrome.

**Figure 4:**
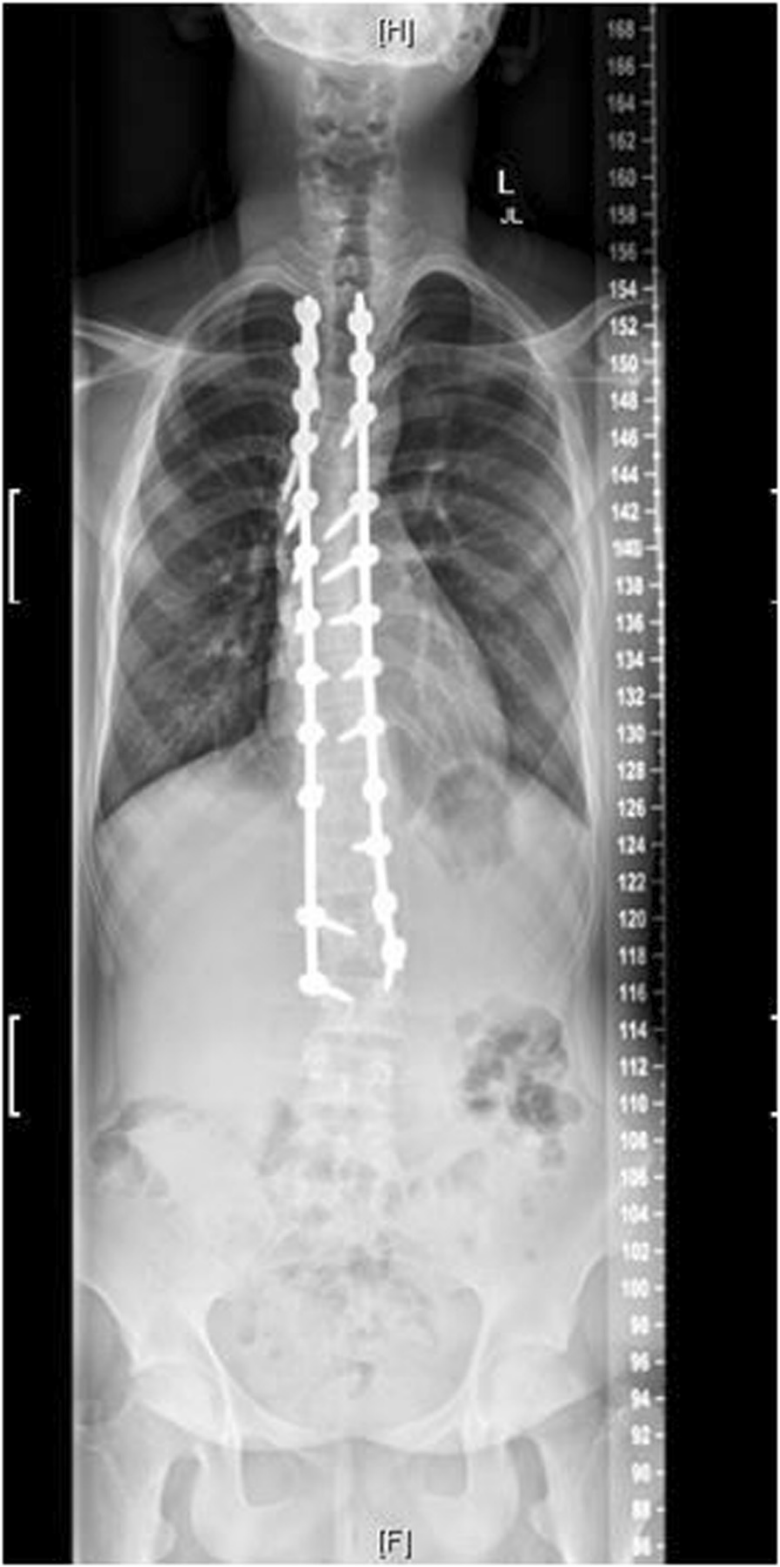
Post-operative radiograph of the same 15 year-old male.

**Figure 5:**
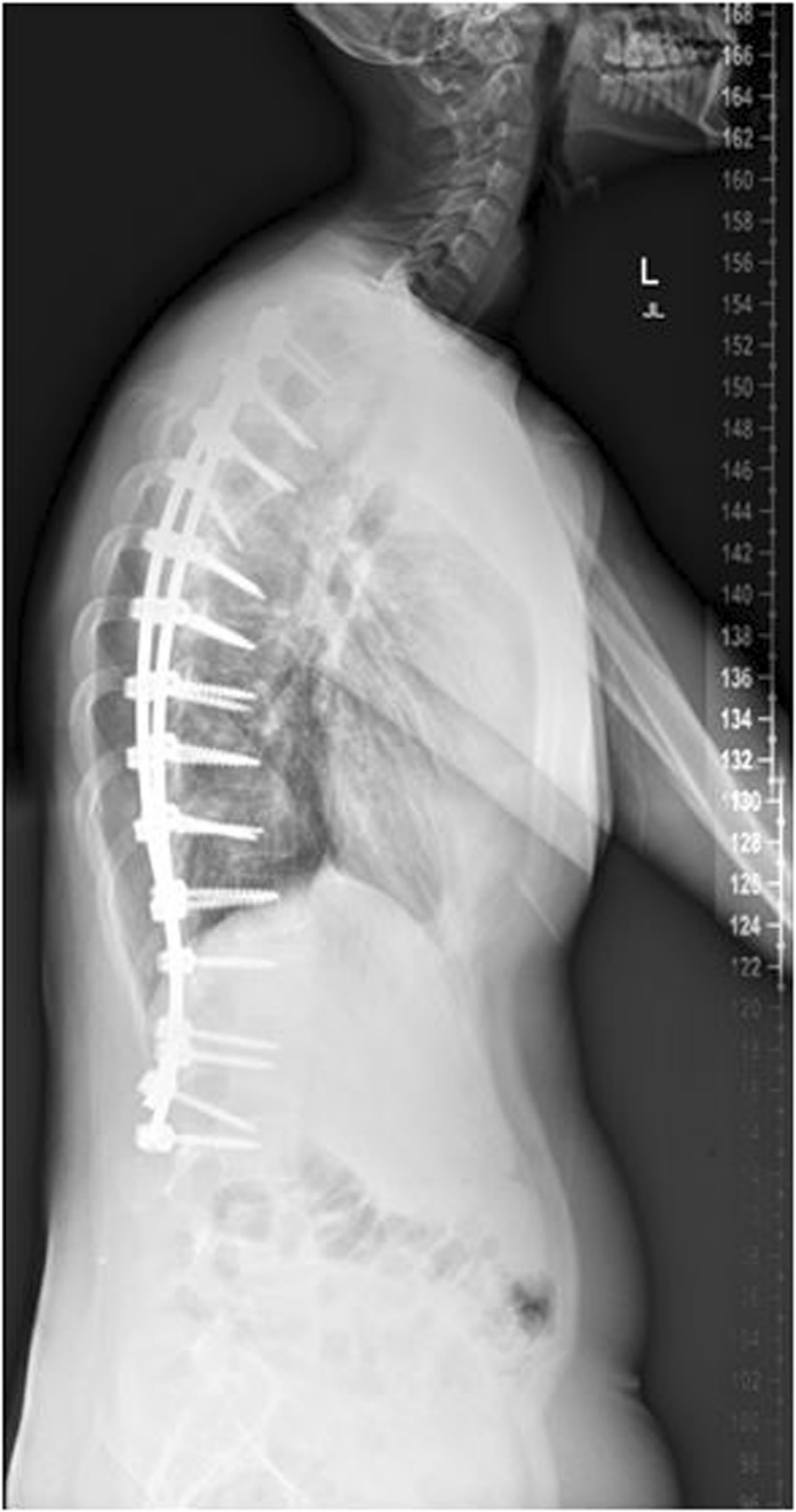
Post-operative radiograph of the same 15 year-old male.

Marfan syndrome, first described in 1896, is the most common syndrome causing spine pathology [4]. Marfan syndrome is an autosomal dominant disease of the connective tissues, resulting from inherited dysfunction of the glycoprotein fibrillin-1 gene on chromosome 15, affecting 1 in 10,000 people [5]. Variability in the fibrillin-1 gene leads to various mutations in the extracellular matrix protein, fibrillin, which aggregates to form microfibrils [6]. Although phenotypic expression can be variable, affected patients often present with skeletal abnormalities including increased height, extremity length, ligamentous laxity, and scoliosis. Deformities of the lens of the eye and cardiovascular abnormalities are also seen. Up to 60% of patients will present with scoliosis, and 40% with kyphosis. Between 25 and 50% of patients have curves significant enough to require surgical intervention, with double or triple major curves appearing more frequently than in other conditions [7,6]. Representative imaging of scoliosis in patients with Marfan syndrome at our institution is demonstrated in Figures 1, 2, 3, 4 and 5.

Six articles were identified that focused on patients with Marfan syndrome (Table 2). Five of these articles examined largely adolescent age patients. One article, by Sponseller et al., focused on infantile patients. A common theme throughout these papers was the high estimated blood loss in these patients, averaging above 1700 mL. Dural tears were another complication commented on frequently. Four articles reported dural tears in 11 of 93 cases. Several articles commented on the need for longer fusion constructs to minimize the risk of implant failure and reoperation [4,5,7-10].

**Table 2:** **Marfan syndrome surgical complications**

	**Dural tear**	**Wound infection**	**Respiratory issues**	**Neuro compromise**	**Hardware failure**	**Pseudarthrosis**	**Avg blood loss mL (#)**
Marfan Pts (# patients reported/total)	11.8% (11/93)	6.5% (6/93)	5.7% (4/70)	2.2% (1/46)	17.2% (20/116)	6.2% (8/129)	1756 (106)

In 2002, Jones et al identified 26 patients receiving spinal fusion for primary scoliosis with Marfan syndrome. The article also included 6 patients undergoing reoperation for progression of scoliosis after prior surgery at another institution. Extension of the fusion to the sacrum was required in 5 of these patients. The authors believed that planning for longer fusion constructs, as well as pre-operative cardiac evaluation should be considered. Furthermore, they noted high blood loss, 2148 mL on average [8]. A second 2002 article, by Lipton et al discussed 23 patients who previously failed brace therapy and underwent instrumented posterior spinal fusion (PSF). High rates of double and triple major curves were found, and the authors emphasized the importance of arthrodesing all primary and secondary curves [5]. A 2005 article by DiSilvestre et al examined 23 patients, and found that distal hook malfunctioning caused loss of correction in 5 of the 23 (21.7%) patients, while 2 of the 23 (8.7%) patients experienced rod fractures leading to revision surgery [4]. Zenner et al collected 23 patients for a 2014 study, echoing the high blood loss, especially in patients undergoing PSF [9].

In 2012, Gjolaj et al compared 34 cases of spinal fusion in Marfan patients to age matched AIS controls and did not find a significant difference in estimated blood loss (EBL).While this differed from the findings of other articles, EBL still averaged 1700 mL for these cases. Of the 9 reoperations noted, 2 were due to the need for proximal extension secondary to curve progression. The authors stressed the importance of longer fusions, greater attention to sagittal balance, and the risk of hardware failure and reoperations (11.8% of patients) [7].

Marfan syndrome may also predispose patients to early onset scoliosis. Sponseller et al investigated this unique population in a series of 10 young patients (mean age 5.3 at time of surgery) with infantile scoliosis treated with growing rod constructs. They found a high rate of CSF leak, 3 out of 10 patients (30%). The authors believe that newer growing rod constructs could lead to improved outcomes in this challenging population [10].

### Down syndrome

**Figure 6:**
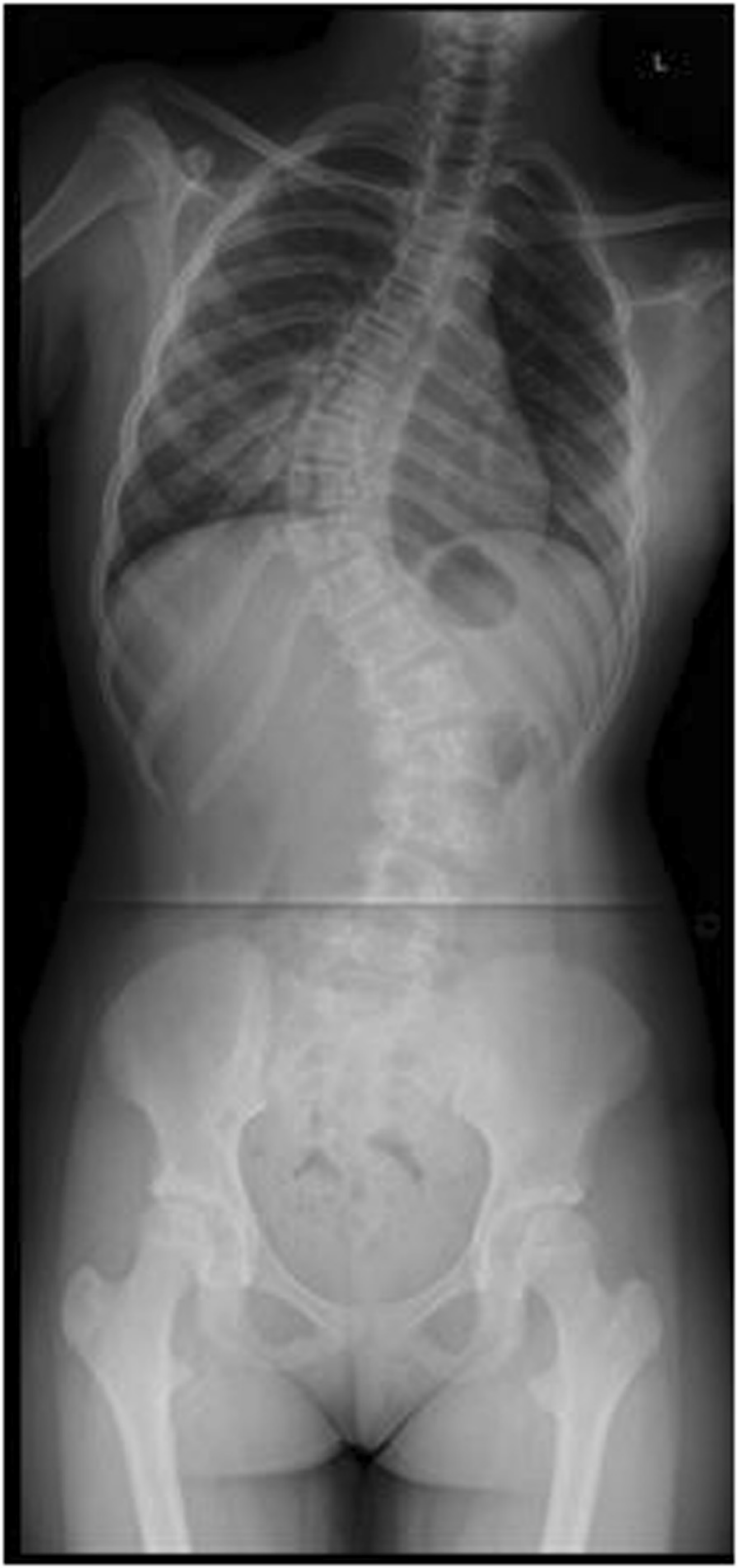
Preoperative radiograph of 12 year-old female with Down syndrome.

**Figure 7:**
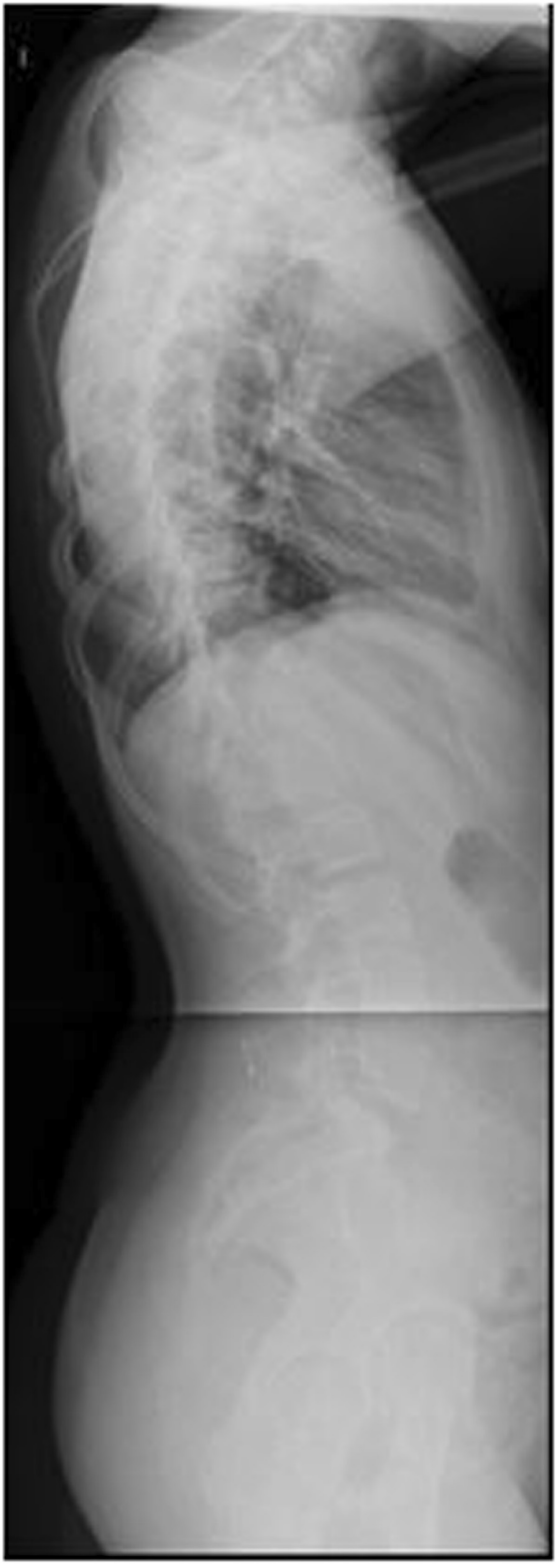
Preoperative radiograph of 12 year-old female with Down syndrome.

**Figure 8:**
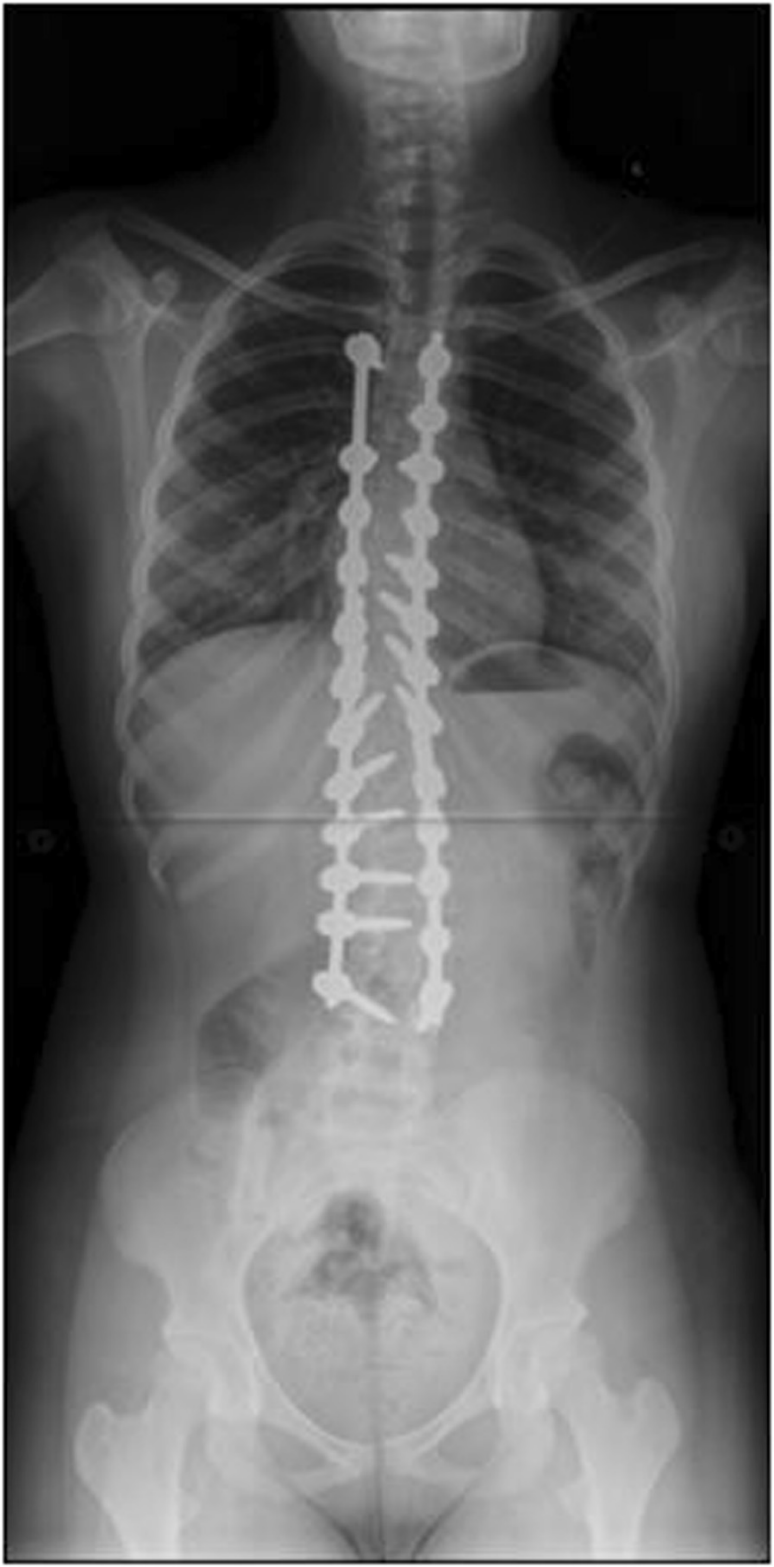
Post-operative image of the same patient at our institution.

**Figure 9:**
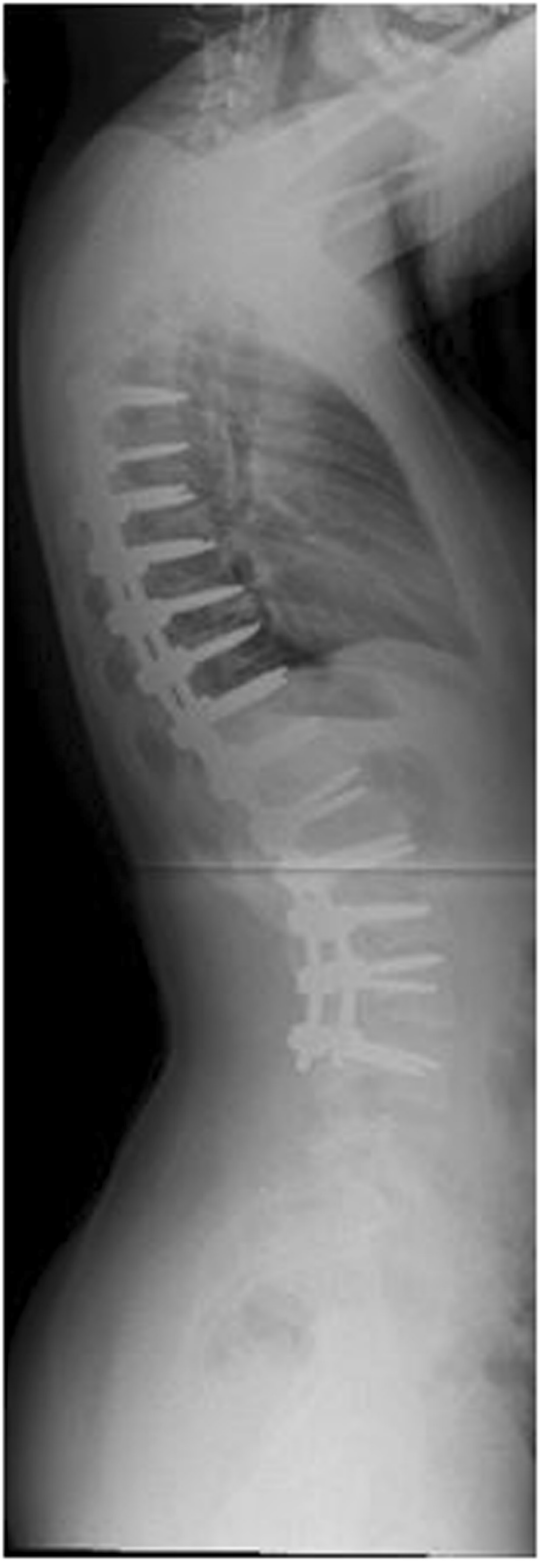
Post-operative image of the same patient at our institution.

Down Syndrome is the most common chromosomal abnormality in the US, and most frequent cause of mental retardation. It stems from trisomy of the 21^st^ chromosome, first identified in 1959 [11]. Prevalence in the US is approximately 1 in 700 live births. Patients surviving through infancy experience cardiac abnormalities, ulcerative colitis, and scoliosis at rates significantly higher than the general population [11]. Clinically, these patients often have hypotonia, hyperlaxity of joints, short stature, and awkward gait. Occipito-atlantoaxial instability is reportedin 15% of these patients. Scoliosis has been found to occur in between 10 and 55% of patients with Down syndrome. Bracing has traditionally been recommended for this group, but with little data regarding outcomes [11,12]. Imaging from a representative patient at our institution with Down syndrome can be viewed in Figures 6, 7, 8 and 9.

Despite the high prevalence of Down syndrome in the general population, only two articles, comprising 14 patients, were found regarding scoliosis surgery complications (Table 3). This may be related to the somewhat discouraging results reported below.

**Table 3:** **Down syndrome surgical complications**

	**Dural tear**	**Wound infection**	**Respiratory issues**	**Neuro compromise**	**Hardware failure**	**Pseudarthrosis**	**Avg blood loss mL (#)**
Down Pts (# patients reported/total)	Not reported	14.3% (2/14)	14.3% (1/7)	Not Reported	35.7% (5/14)	28.6% (4/14)	1264 (7)

In 2003, Lerman et al examined 7 patients with Down Syndrome and scoliosis operated on over a 16 year period. The authors found a high rate of overall complications (71.4%), with one case each of wound infection (requiring return to operating room for washout), pneumonia, hardware failure, and psuedarthrosis. The authors also noted two episodes (28.6%) of delayed wound healing without infection [13]. A second article from 2005 by Milbrandt et al, studying patients from one institution treated over a 50 year period identified just 7 patients operated on for scoliosis with Down syndrome out of 33 patients with scoliosis. Four of the seven (57.1%) cases had failure of surgical implants, a significantly high rate compared to other syndromes. Approximately half of patients studied had already had cardiac surgery, a comorbidity associated with Down syndrome. The authors indicated that the complications incurred were significant enough to have “overshadowed” the benefits of the surgery [11].

### Rett syndrome

Rett syndrome is a progressive neuroaffective disorder occurring almost exclusively in females [14]. The syndrome is caused by a mutation of the MECP2 gene on the X chromosome [15]. Symptoms begin to present around the age of one year. Criteria for clinical diagnosis include disruption of purposeful hand skills, stereotyped hand movements, decelerated head growth, communication dysfunction, failure to move, and neuromotor regression. Scoliosis is present in this population significantly above the general population, with estimates anywhere between 36% and 100% of the affected individuals [14]. The incidence of scoliosis in this population increases with age and often present in a sweeping, “C-shape” pattern [16].

All articles about Rett syndrome discussed pulmonary complications (Table 4). Almost 30% of patients were reported as having respiratory complications, including pneumothorax, respiratory failure, and pneumonia. Despite the complications, and in contradistinction to many reports on Down syndrome patients, the authors generally felt that the surgeries were successful in bettering the overall lives of these patients [14,16,17].

**Table 4:** **Rett syndrome surgical complications**

	**Dural tear**	**Wound infection**	**Respiratory issues**	**Neuro compromise**	**Hardware failure**	**Pseudarthrosis**	**Avg blood loss mL (#)**
Rett Pts (# patients reported/total)	Not reported	10.1% (9/89)	29.2% (26/89)	Not Reported	4.5% (3/66)	0% (0/16)	2106 (16)

In 2003 Kerr et al reported on 50 patients with Rett syndrome. They found a 20% rate of pulmonary complications, including six cases of collapsed lungs, and two patients that required ventilator support post-operatively. “Movement of stabilizing rods” caused reoperation in 2 of the patients. 72% of patients were unable to ambulate prior to or after the surgery, and one patient who walked before surgery could not afterwards. Despite complications, the authors indicated that 84% of patients reported improvement in their well-being, despite worsening of some elements, such as toileting. Importantly, the authors also stressed the importance of family involvement with care and rehabilitation as well as timing of surgery and nutrition during the peri-operative time period [17]. In 2009, Larsson et al reviewed 23 patients, again stressing respiratory complications, 26.1% of patients, 2 patients requiring extended respiratory treatment. The authors felt that the surgery was beneficial in allowing for more adequate breathing in these patients, preventing future episodes of pneumonia [16]. Gabos et al, in 2012, reported on 16 patients; with average blood loss of 2106 mL, mentioned to be notably high. Respiratory concerns were stressed, with 6 cases of pneumonia, 3 cases of pneumothorax, and two patients needed chest tubes placed. The article also noted “major” GI complications in 37% of patients and at least “minor” GI complications in every patient studied [14].

### Neurofibromatosis

**Figure 10:**
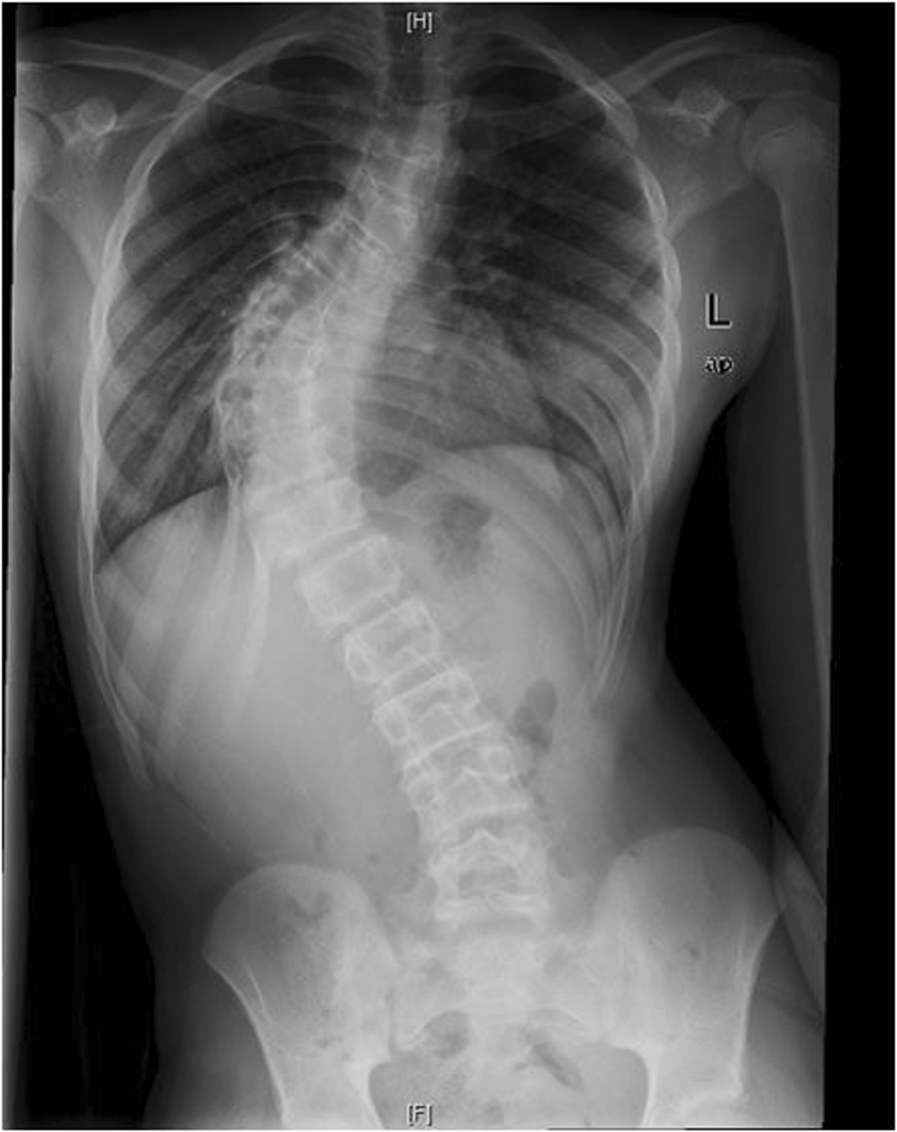
Preoperative radiograph of 12 year-old female with NF.

**Figure 11:**
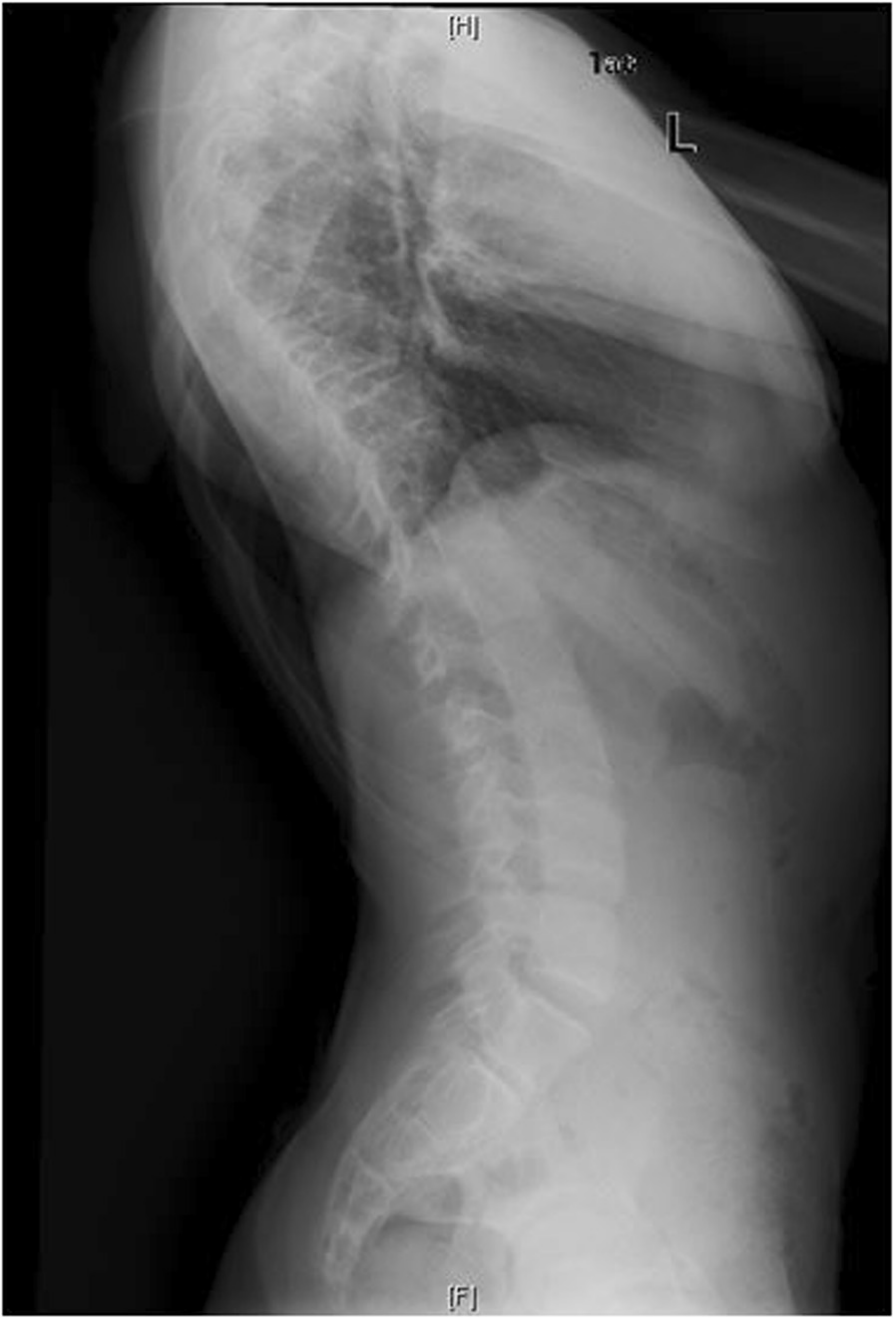
Preoperative radiograph of 12 year-old female with NF.

**Figure 12:**
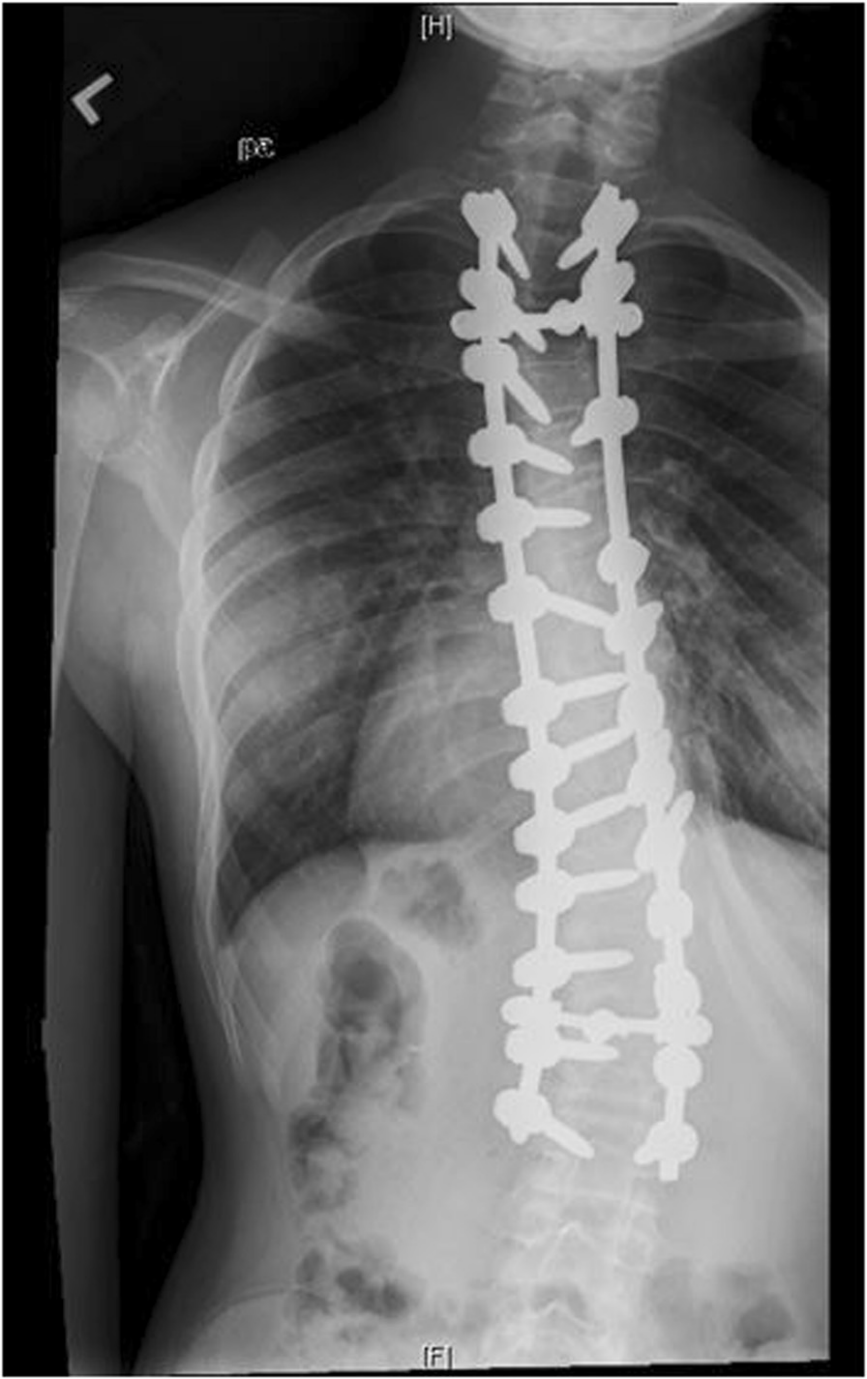
Post-operative image of the same patient.

**Figure 13:**
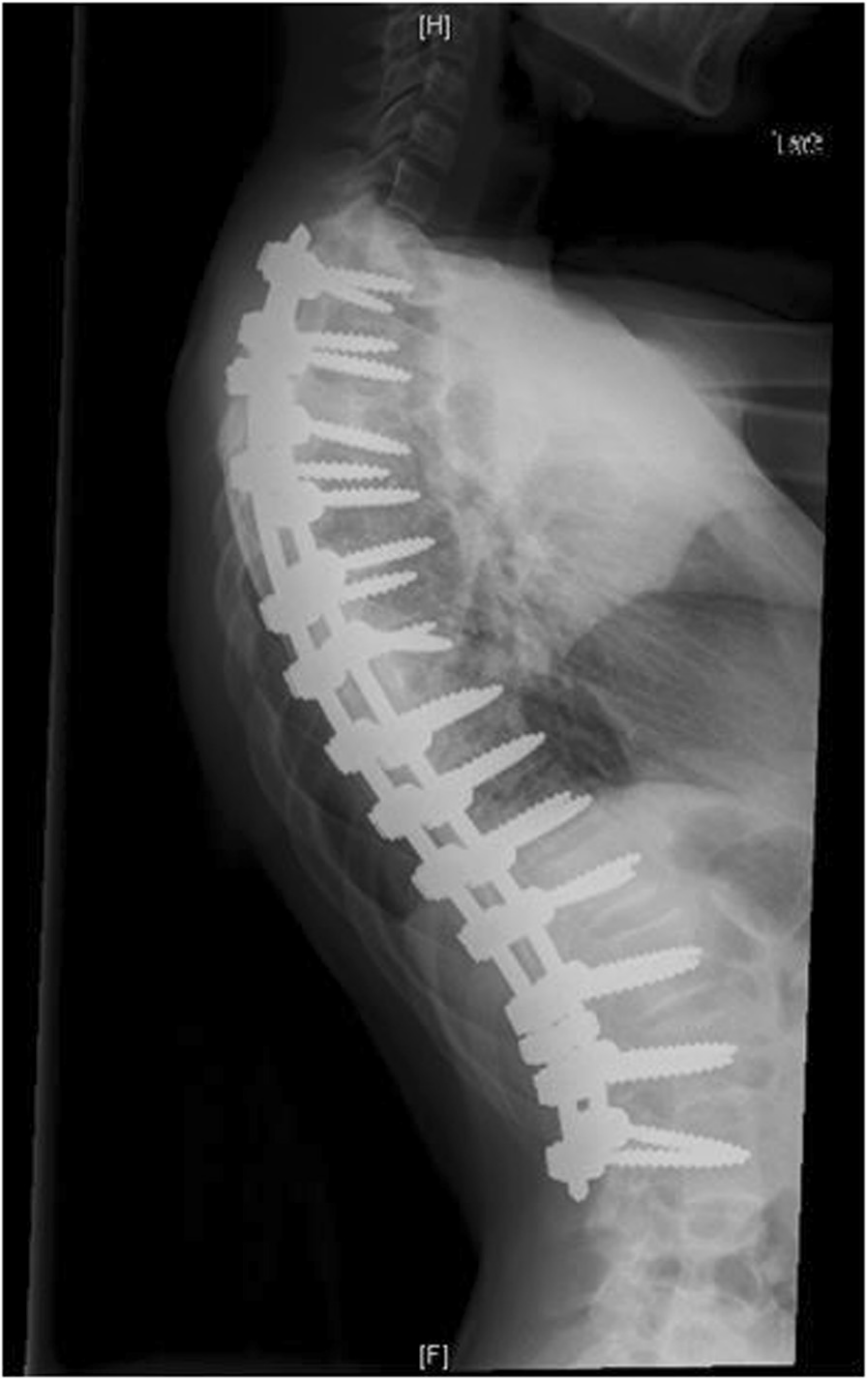
Post-operative image of the same patient.

Neurofibromatosis (NF) is among the most common single gene mutations in the general population with a prevalence of 1 in 3000 people [18,19]. There are two main types of the disorder, peripheral or type 1, and central or type 2. Scoliosis has been reported in both types [18]. A defect in the NF1 gene on chromosome 17q11.2 leads to NF type 1 or von Recklinghausen’s Disease, first identified in the late 19th century [20]. Phenotypes are variable, ranging from no discernible symptoms to disfiguring spinal deformities with disruption of vital organs and the spinal cord itself. While sharp, angular, “dystrophic” curves are typically associated with NF, mny patients have curves more typical of AIS “non-dystrophic.” Both curve patterns tend to progress more rapidly than AIS, but dystrophic curves are more characteristic of the disease and can be particularly aggressive. Surgical treatment has been reported to be problematic secondary to dural ectasias, and poor bone stock [18,21]. Furthermore, the progressive nature of deformities in some patients has led to curve progression even after arthrodesis [22]. Imaging from a representative patient at our institution with NF can be viewed in Figures 10, 11, 12 and 13.

Overall, five studies were identified regarding NF (Table 5). Three reported a combined 10% rate of wound infections, a complication particularly notable in this population. Additionally, 5 out of 88 patients (5.7%) experienced neurological complications related to surgery. Several articles noted the progression of curves following surgery, especially in the dystrophic subtype- in some cases leading to reoperation [18,19,21-23].

**Table 5:** **Neurofibromatosis surgical complications**

	**Dural tear**	**Wound infection**	**Respiratory issues**	**Neuro compromise**	**Hardware failure**	**Pseudarthrosis**	**Avg blood loss mL (#)**
NF Pts (# patients reported/total)	5.9% (3/51)	10.2% (9/88)	Not reported	5.7% (5/88)	4.2% (4/96)	5.7% (5/88)	903 (51)

Wilde et al in 1994 studied 25 patients, reporting wound infections in 24% of patients, 33% of these infections required return to the operating room for surgical debridement. The authors highlighted the worsening of curves postoperatively in some patients undergoing surgical correction, even in those with solid arthrodesis [22]. Halmai et al. [19] stressed the importance of early detection and intervention, given the rapidly progressive nature of the deformity. The authors noted one case of paraplegia discovered intraoperatively due to a neurofibroma penetrating into the spinal cord [19]. Shen et al in 2005 indicated that 13.3% of patients required reoperation, a complication that patients should be prepared for [23]. A 2009 article by Li et al. found that 3 out of 16 (18.8%) patients that they operated on with dystrophic curves had intraoperative dural tears. The authors also noted the commonality of post-operative curve progression in this population, attributed to poor bone stock, especially in dystrophic curves [21]. Koptan et al. [18], found wound infections in 9.4% of patients, only one of which required return to the OR for debridement.

### Ehlers-Danlos

Ehlers-Danlos syndrome, first described by Ehlers and Danlos in the early 20th century [24], is caused by a malformation of type III collagen, affecting up to 1 in 5000 individuals [25]. The skin, ligaments, and joints are particularly lax. Some individuals with Ehlers-Danlos may be affected by congenital scoliosis, hypotonia, and ocular fragility. A deficiency of lysyl hydroxylase, an enzyme that modifies collagen, leads to joint laxity and muscle hypotonia [25]. Scoliosis or kyphoscoliosis in these patients generally occurs in the thoracic or lumbar spines and may appear shortly after birth. Surgery correcting these deformities has previously been described as having significant complications [26].

Three papers were identified that focused on Ehlers-Danlos patients (Table 6). The authors of each paper noted a significant bleeding risk in this patient population. All 3 articles emphasized blood loss measurements, which averaged 1272 mL intraoperatively. One reported death postoperatively from vascular compromise was discussed as well [24,26,27].

**Table 6:** **Ehlers-Danlos syndrome surgical complications**

	**Dural tear**	**Wound infection**	**Respiratory issues**	**Neuro compromise**	**Hardware failure**	**Pseudarthrosis**	**Avg blood loss mL (#)**
ED Pts (# patients reported/total)	16.7% (1/6)	Not reported	20% (1/5)	0% (0/16)	22.7% (5/22)	36.3% (4/11)	1272 (22)

Akpinar et al. [24] reported an average blood loss of 1764 mL in five patients with Ehlers-Danlos. Two episodes of intraoperative vascular complications were reported, including a ruptured iliac artery in one case. The authors commented on the significant bleeding risk in these patients secondary to vascular fragility. They recommended special attention to detail regarding bleeding, including the potential benefits of hypotensive anesthesia [24]. Jasiewicz et al. [26] found that 4 of 11 patients treated for their scoliosis required reoperation secondary to imbalance and instrumentation failure. Rabenhorst et al. [27], looked at 6 patients with Ehlers-Danlos, reported that one patient died following PSF secondary to intra-abdominal bleeding. The authors also noted the need for removal of hardware over one year postoperatively secondary to infection in two patients [27].

### Osteogenesis imperfecta

Osteogenesis Imperfecta (OI) stems from a disruption in type I collagen, affecting approximately 1 in 10,000 live births [28]. Aside from bone, structural changes are seen in the skin, eyes, ears, and teeth. The most severe form is incompatible with life beyond infancy, others may be minimally affected. Most common, though not universal, presentations include blue sclera and hearing loss in young patients in addition to bone fragility. Scoliosis in this population is often progressive into adulthood, and associated with chest wall abnormalities leading to compromised pulmonary function. The excessive fragility in these patients causes increased difficulty in surgery [29].

Two articles were comprised of patients with OI, both noting the significant disabilities, even postoperatively, in this population (Table 7). Janus et al. [29] collected data from 20 patients with OI, noting one episode of deep wound infection and one episode of neurological compromise. The authors indicated that this is a difficult population, stating that patients had increased functioning in only 35% of cases, compared with a 20% rate of overall complications [29]. Topouchian et al. [30] reported that a rod fracture leading to loss of correction occured in 14.8% of their patient group.

**Table 7:** **Osteogenesis imperfecta surgical complications**

	**Dural tear**	**Wound infection**	**Respiratory issues**	**Neuro compromise**	**Hardware failure**	**Pseudarthrosis**	**Avg blood loss mL (#)**
OI Pts (# patients reported/total)	Not reported	5.0% (1/20)	Not Reported	5% (1/20)	12.8% (6/47)	5 % (1/20)	Not Reported

### Prader-Willi

**Figure 14:**
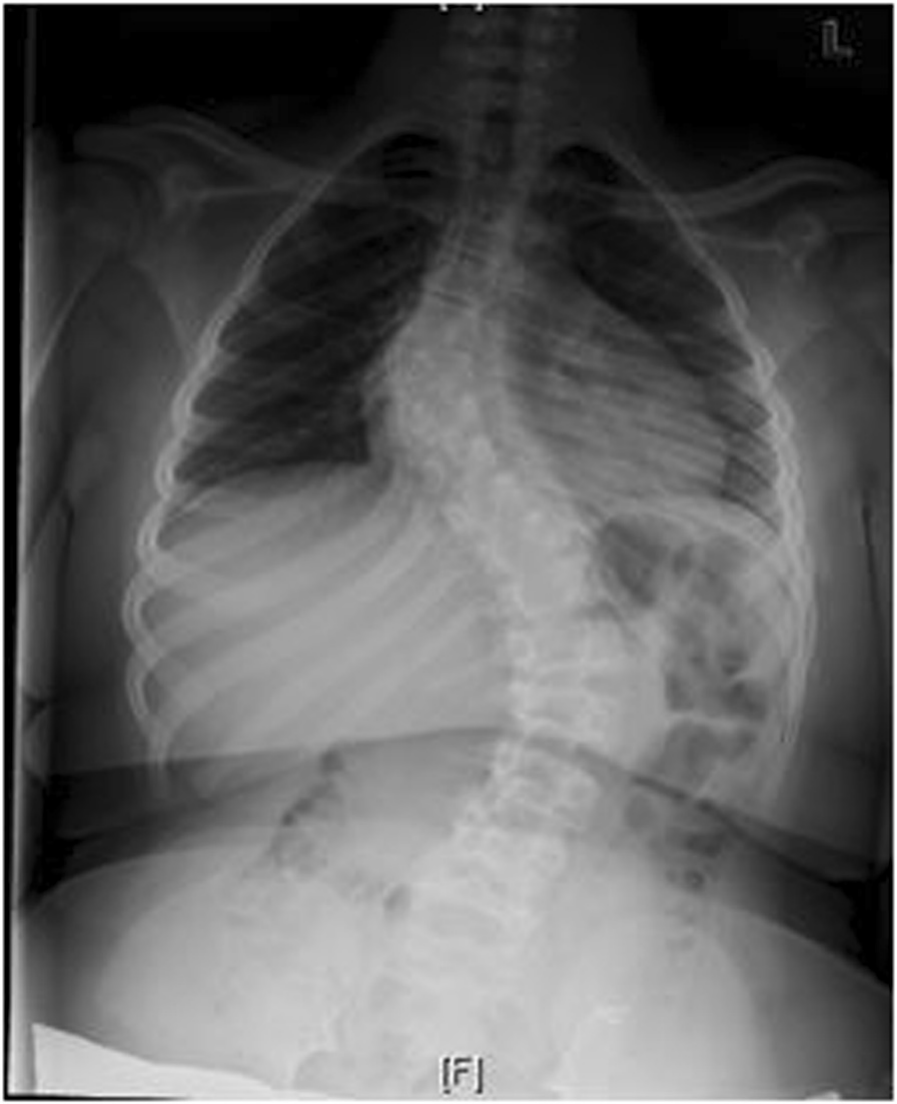
Preoperative radiograph of a 14 year old with Prader-Willi syndrome.

**Figure 15:**
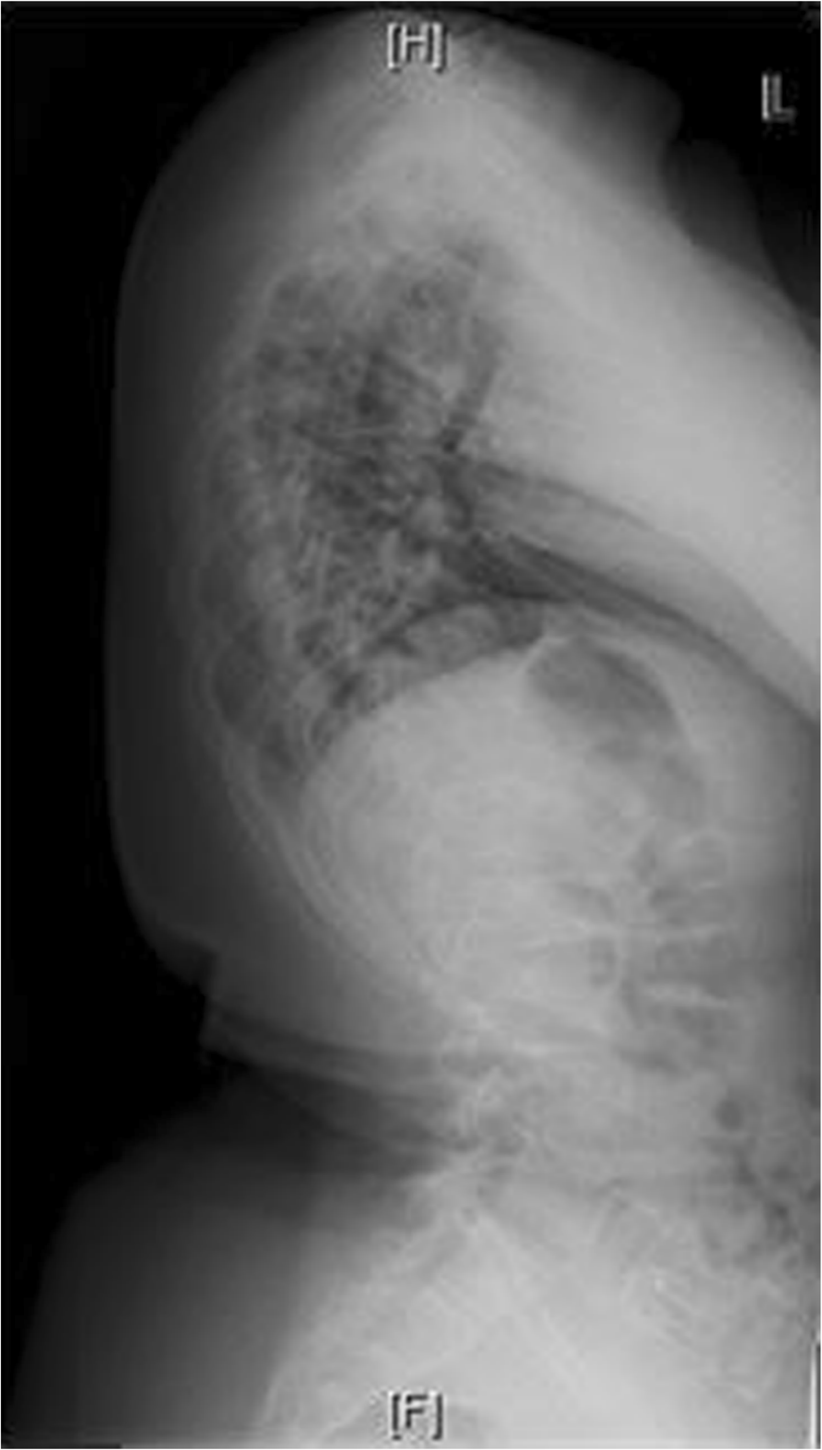
Preoperative radiograph of a 14 year old with Prader-Willi syndrome.

**Figure 16:**
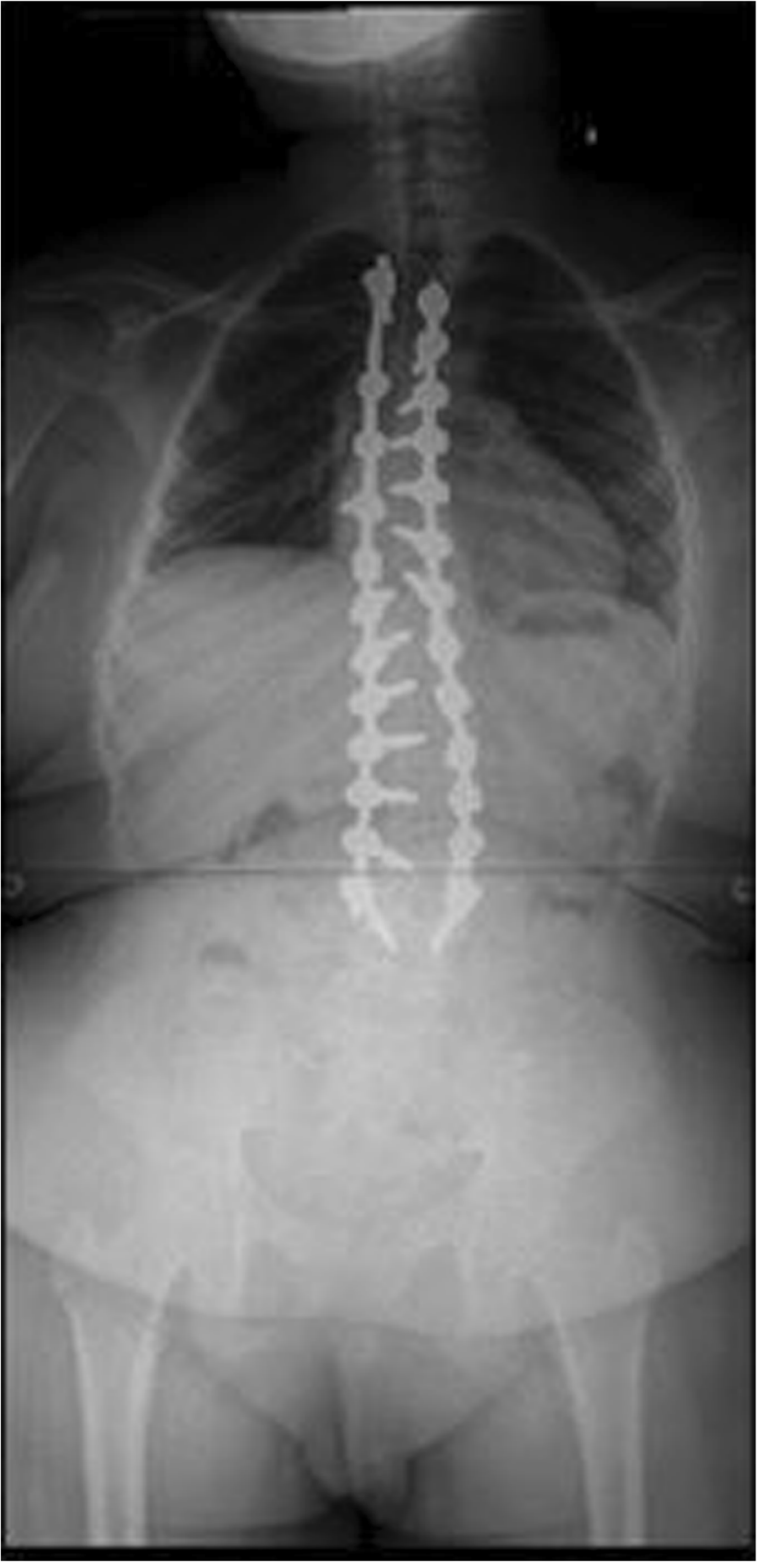
Post-operative image of the same patient.

**Figure 17:**
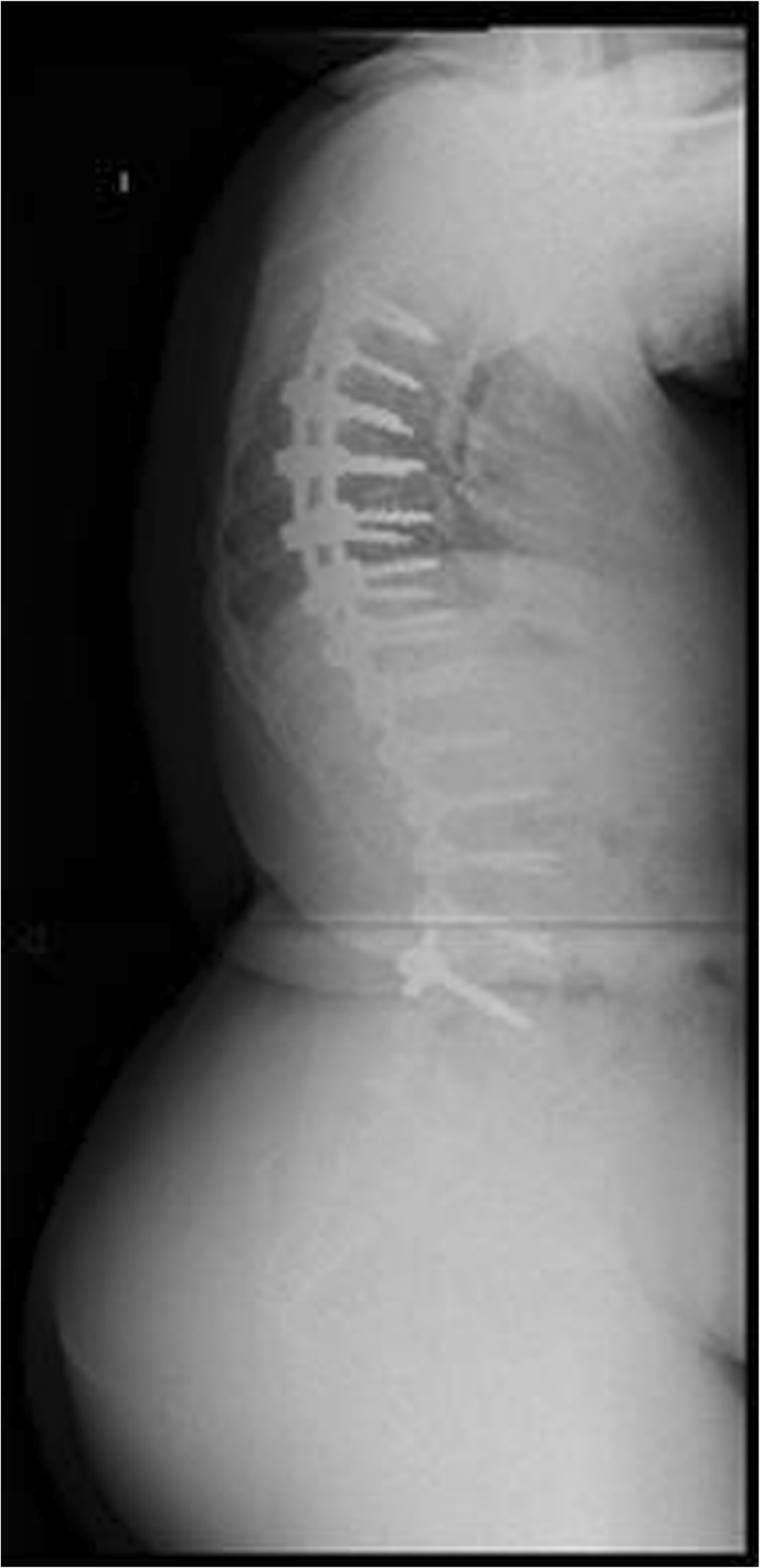
Post-operative image of the same patient.

Prader-Willi syndrome, first described in 1956, [31] has a significantly variable presentation, but has been associated most commonly with mental retardation, short stature, obesity, hypogonadism, and hypotonia, as well as scoliosis. Some believe that hypotonia and subsequent muscle weakness lead to the kyphoscoliosis which may present up until adolescence [32]. The condition is caused by a deletion in the parental copy of the 15q11.2 chromosome, affecting approximately 1 in 15,000 infants. Most estimates indicate that half or more of patients with Prader-Willi have scoliosis [33]. One study demonstrated that the rate of scoliosis in patients with Prader-Willi consistently increased with age up until the point of skeletal maturity, affecting approximately 2/3 of skeletally mature individuals. Furthermore, the characteristic obesity associated with the condition appears to contribute to the development of kyphosis [34]. Imaging from a representative patient at our institution with Prader-Willi syndrome can be viewed in Figures 14, 15, 16 and 17.

Two articles focusing on Prader-Willi syndrome were identified, both emphasizing episodes of neurological compromise (Table 8). Accadbled et al. [31], found that of 16 patients with PW, 5 had wound infections, with need for irrigation and debridement in 3. Four patients had neurological compromise, including definitive tetraplegia in 1, and definitive paraplegia in another. The authors concluded that the surgical risk may outweigh the potential benefits [31]. Greggi et al. [33], examined 6 cases aged 10-15 in their paper, showing an average blood loss of 1750 mL. The authors felt that bracing was less effective in these patients than AIS patients, and surgery is thus more frequently necessary. Given the higher rates of neurological issues in these patients, including one episode of transient paraparesis, the authors suggested preoperative spine MRI and intraoperative spinal cord monitoring to reduce complications [33].

**Table 8:** **Prader-Willi syndrome surgical complications**

	**Dural tear**	**Wound infection**	**Respiratory issues**	**Neuro compromise**	**Hardware failure**	**Pseudarthrosis**	**Avg blood loss mL (#)**
PW Pts (# patients reported/total)	Not reported	22.7% (5/22)	6.3% (1/16)	22.7% (5/22)	22.7% (5/22)	4.5% (1/22)	1750 (6)

### Friedreich’s Ataxia

Friedreich’s Ataxia is a progressive disorder characterized by spinocerebellar degeneration over time, affecting approximately 1 in 50,000 individuals [35]. Symptoms include progressive impairment of pain, temperature, vibratory, and light touch sensation, as well as ambulation impairment leading to wheelchair necessity. Scoliosis, which often does not present until adolescence, occurs, in some estimations, in up to 100% of this population. This disorder is caused by an expansion repeat of the GAA trinucleotide on the 9q13 chromosome, leading to iron deposition in the mitochondria and ultimately creation of free radicals [35]. Cardiomyopathy leads to death in many patients before the age of 40 [36].

A single 2008 study by Milbrandt et al discussed 16 patients (mean age 15.5) with Friedreich’s ataxia (Table 9). The authors noted one episode of deep wound infection following surgery, which required implant removal and reoperation for washout. They reported that 81% of operated-on patients remained wheelchair-bound at surgical follow-up. Moreover, they concluded that there was no significant impact from surgical correction on what age patients became wheelchair bound. Despite cardiomyopathy concerns, the authors felt that surgery could be preformed safely with close anesthesia monitoring [36].

**Table 9:** **Friedreich’s ataxia surgical complications**

	**Dural tear**	**Wound infection**	**Respiratory issues**	**Neuro compromise**	**Hardware failure**	**Pseudarthrosis**	**Avg blood loss mL (#)**
FA Pts (# patients reported/total)	Not reported	6.3% (1/16)	6.3% (1/16)	0% (0/16)	6.3% (1/16)	Not Reported	1268 (16)

## Discussion

Syndromic scoliosis represents a wide range of underlying pathologies and phenotypic manifestations. This paper is a thorough analysis of the recent literature concerning the quantitative and qualitative complication rates associated with these challenging patients. To provide a reference point for our data, a comparison population is necessary. Adolescent Idiopathic Scoliosis (AIS) is the most studied and published-upon category of scoliosis. Furthermore, given the high rate of AIS patients operated on at most institutions, this population’s data provides an excellent tool for comparison (Table 10).

**Table 10:** **Adolescent idiopathic scoliosis surgical complications**

	**Dural tear (Durotomy)**	**Wound infection (Deep and Superficial)**	**Respiratory issues (Pulmonary) + (PE)**	**Neuro compromise (New Neuro Compromise) + (Peripheral Nerve)**	**Hardware malfunction (Implant Related)**	**Pseudarthrosis**	**Avg blood loss mL (#)**
AIS Pts (# patients reported/total)	0.2% (22/11,227)	1.4% (156/11,227)	0.6% + 0.04% (63/11,227 + 5/11,227)	0.8% + 0.5% (86/11,227 + 53/11,227)	1.1% (120/11,227)	Not reported	323-907-1277

A 2011 study by Reames et al looked into the surgical complications associated with the repair of over 19,000 patients with scoliosis. The complications analyzed were divided into the various main etiologies of scoliosis: idiopathic, congenital, neuromuscular, and other (comprised of post-traumatic, “syndromic”, neurofibromatosis, non-neurologic tumor, iatrogenic, and bone dysplasia/dwarfism). The most represented group, “Idiopathic”, encompassed over 11,000 patients and had complication rates as follows: 6.3% of patients had complications of any kind, 0.8-1.3% of patients had wound infections, and pulmonary complications, durotomies, and neurologic complications occurred in less than 1% of cases [37].

Of note, Reames also discussed complications in a group referred to as “Other” which included syndromic causes of scoliosis. This group was not stratified by specific syndrome, and little data was elaborated upon. Thus, the issue of heterogeneity between different syndromes comprising the syndromic group was not addressed and stratified, as it was in our analysis.

Blood loss is one of the most common and significant complications reported in syndromic scoliosis patients. In 2013, Ialenti et al reported on a cohort of AIS patients and found that estimated blood loss ranged from 907 +/-775 mL for those treated with posterior spinal fusion (PSF), to 323+/-171 mL in those treated with anterior spinal fusion (ASF), to 1277+/- 821 mL in a limited number of combined-approach cases. While a large variability was found from study to study, the results of our analysis indicate that average blood loss in syndromic patients is significantly higher, regardless of surgical approach. Notably, in Marfan and Rett Syndrome patients EBL is approximately 2000 mL per case, significantly higher than that of AIS patients [38].

### Complication rates in surgically treated AIS patients from two recent studies [37,38]

Lung disease was also a commonly reported specific complication that was seen throughout our analysis. A recent paper by McPhail et al, noted that 6 out of 18 patients with syndromic scoliosis had obstructive lung disease, while 57% suffered from restrictive lung disease [39]. This data supports the notion reported above that patients with syndromic scoliosis have additional comorbidities, including respiratory issues preoperatively. This may be contributory to the high rates of respiratory issues we noted perioperatively in our data set.

A 2013 meta-analysis by Sharma et al focused on the neuromuscular scoliosis population, examining 68 articles between 1997 and 2011. They found that 22.7% of patients had pulmonary complications, 12.5% had implant complications, 10.9% had wound infections, 3% had neurological complications, and 1.9% had pseudarthrosis [3]. These rates, though somewhat variable, are largely similar to the results found in our article.

## Conclusion

The complication profiles that we report in this paper are significantly higher than in patients with AIS and at least comparable to those seen in neuromuscular scoliosis [3,37,38].

There are unique management issues that are relevant when discussing the patients in the syndromic group beyond the parameters that were identified in the study presented in this paper. For example, the issue of dealing with patients with mental handicaps (common in many of the syndromes included under the umbrella of syndromic scoliosis) presents added challenges to rehabilitation and repair following surgery. The solution to minimizing risk in these patients will likely include a multidisciplinary approach, integrating family, nursing, anesthesia, and other providers in the role of patient care. For example, given the high rates of pulmonary complications in patients with Rett Syndrome, the importance of integrative perioperative pulmonary care cannot be overstated. Fluid resuscitation and transfusion protocols are also important, given the increased blood loss detailed in our study. It is hoped that added attention to detail, specifically with an interdisciplinary approach, more predictable and safer surgeries can be achieved.

Given the rarity of each of these syndromic cases, establishing a national network in which we can track outcomes and research is necessary. Providing more data should allow for the development of protocols tailored to the needs of these patients, and can serve as a model for improving the care of patients with other rare spinal abnormalities or medical conditions.

While this analysis has many limitations, perhaps chief among them is that the articles studied ranged over three decades. Surgical practice is constantly evolving, and thus techniques and technology (surgical, anesthetic, perioperative) will change dramatically. We acknowledge that the differences in countries of study, surgical approach, surgical technique, and others represent confounding variables.

Furthermore, while most of the complications occurred early in the post operative period, given the different reporting characteristics of each paper we have been unable to stratify complications consistently according to whether they were early, intermediate or late surgical complications.

Although the complication rates for syndromic scoliosis surgery are significant, most authors believed that the surgeries themselves were overall beneficial. One notable exception is Down syndrome in which both papers reported disappointing results. Given the potential rewards of surgery and the high level of complications in patients with syndromic scoliosis, it is essential to examine the quantity and quality of these complications to pinpoint the source of intraoperative and postoperative challenges. This analysis is a step forward in consolidating and considering these complications in syndromic scoliosis.

### Consent

Written informed consent was obtained from the patient for the publication of this report and any accompanying images. The patients allowed their medical history and imaging to be published.

## References

[1] Edler A, Murray DJ, Forbes RB (2003). Blood loss during posterior spinal fusion surgery in patients with neuromuscular disease: is there an increased risk?. Paediatr Anaesth.

[2] Hassan N, Halanski M, Wincek J, Reischman D, Sanfilippo D, Rajasekaran S (2011). Blood management in pediatric spinal deformity surgery: review of a 2-year experience. Transfusion.

[3] Sharma S, Wu C, Andersen T, Wang Y, Hansen ES, Bunger CE (2013). Prevalence of complications in neuromuscular scoliosis surgery: a literature meta-analysis from the past 15 years. Eur Spine J.

[4] Di Silvestre M, Greggi T, Giacomini S, Cioni A, Bakaloudis G, Lolli F (2005). Surgical treatment for scoliosis in Marfan syndrome. Spine (Phila Pa 1976).

[5] Lipton GE, Guille JT, Kumar SJ (2002). Surgical treatment of scoliosis in Marfan syndrome: guidelines for a successful outcome. J Pediatr Orthop.

[6] Ramirez F, Dietz HC (2007). Marfan syndrome: from molecular pathogenesis to clinical treatment. Curr Opin Genet Dev.

[7] Gjolaj JP, Sponseller PD, Shah SA, Newton PO, Flynn JM, Neubauer PR (2012). Spinal deformity correction in Marfan syndrome versus adolescent idiopathic scoliosis: learning from the differences. Spine (Phila Pa 1976).

[8] Jones KB, Erkula G, Sponseller PD, Dormans JP (2002). Spine deformity correction in Marfan syndrome. Spine (Phila Pa 1976).

[9] Zenner J, Hitzl W, Meier O, Auffarth A, Koller H (2014). Surgical outcomes of scoliosis surgery in marfan syndrome. J Spinal Disord Tech.

[10] Sponseller PD, Thompson GH, Akbarnia BA, Glait SA, Asher MA, Emans JB (2009). Growing rods for infantile scoliosis in Marfan syndrome. Spine (Phila Pa 1976).

[11] Milbrandt TA, Johnston CE (2005). Down syndrome and scoliosis: a review of a 50-year experience at one institution. Spine (Phila Pa 1976).

[12] Antonarakis SE, Lyle R, Dermitzakis ET, Reymond A, Deutsch S (2004). Chromosome 21 and down syndrome: from genomics to pathophysiology. Nat Rev Genet.

[13] Lerman JA, Emans JB, Hall JE, Karlin LI (2003). Spinal arthrodesis for scoliosis in Down syndrome. J Pediatr Orthop.

[14] Gabos PG, Inan M, Thacker M, Borkhu B (2012). Spinal fusion for scoliosis in Rett syndrome with an emphasis on early postoperative complications. Spine (Phila Pa 1976).

[15] Amir RE, Van den Veyver IB, Wan M, Tran CQ, Francke U, Zoghbi HY (1999). Rett syndrome is caused by mutations in X-linked MECP2, encoding methyl-CpG-binding protein 2. Nat Genet.

[16] Larsson EL, Aaro S, Ahlinder P, Normelli H, Tropp H, Oberg B (2009). Long-term follow-up of functioning after spinal surgery in patients with Rett syndrome. Eur Spine J.

[17] Kerr AM, Webb P, Prescott RJ, Milne Y (2003). Results of surgery for scoliosis in Rett syndrome. J Child Neurol.

[18] Koptan W, ElMiligui Y (2010). Surgical correction of severe dystrophic neurofibromatosis scoliosis: an experience of 32 cases. Eur Spine J.

[19] Halmai V, Doman I, de Jonge T, Illes T (2002). Surgical treatment of spinal deformities associated with neurofibromatosis type 1. Report of 12 cases. J Neurosurg.

[20] Ferner RE, Gutmann DH (2013). Neurofibromatosis type 1 (NF1): diagnosis and management. Handb Clin Neurol.

[21] Li M, Fang X, Li Y, Ni J, Gu S, Zhu X (2009). Successful use of posterior instrumented spinal fusion alone for scoliosis in 19 patients with neurofibromatosis type-1 followed up for at least 25 months. Arch Orthop Trauma Surg.

[22] Wilde PH, Upadhyay SS, Leong JC (1994). Deterioration of operative correction in dystrophic spinal neurofibromatosis. Spine (Phila Pa 1976).

[23] Shen JX, Qiu GX, Wang YP, Zhao Y, Ye QB, Wu ZK (2005). Surgical treatment of scoliosis caused by neurofibromatosis type 1. Chin Med Sci J.

[24] Akpinar S, Gogus A, Talu U, Hamzaoglu A, Dikici F (2003). Surgical management of the spinal deformity in Ehlers-Danlos syndrome type VI. Eur Spine J.

[25] Mao J-R, Bristow J (2001). The Ehlers-Danlos syndrome: on beyond collagens. J Clin Invest.

[26] Jasiewicz B, Potaczek T, Tesiorowski M, Lokas K (2010). Spine deformities in patients with Ehlers-Danlos syndrome, type IV - late results of surgical treatment. Scoliosis.

[27] Rabenhorst BM, Garg S, Herring JA (2012). Posterior spinal fusion in patients with Ehlers-Danlos syndrome: a report of six cases. J Child Orthop.

[28] Martin E, Shapiro JR (2007). Osteogenesis imperfecta: epidemiology and pathophysiology. Curr Osteoporos Rep.

[29] Janus GJ, Finidori G, Engelbert RH, Pouliquen M, Pruijs JE (2000). Operative treatment of severe scoliosis in osteogenesis imperfecta: results of 20 patients after halo traction and posterior spondylodesis with instrumentation. Eur Spine J.

[30] Topouchian V, Finidori G, Glorion C, Padovani JP, Pouliquen JC (2004). Posterior spinal fusion for kypho-scoliosis associated with osteogenesis imperfecta: long-term results. Rev Chir Orthop Reparatrice Appar Mot.

[31] Accadbled F, Odent T, Moine A, Chau E, Glorion C, Diene G (2008). Complications of scoliosis surgery in Prader-Willi syndrome. Spine (Phila Pa 1976).

[32] Chen C, Visootsak J, Dills S, Graham JM (2007). Prader-Willi syndrome: an update and review for the primary pediatrician. Clin Pediatr (Phila).

[33] Greggi T, Martikos K, Lolli F, Bakaloudis G, Di Silvestre M, Cioni A (2010). Treatment of scoliosis in patients affected with Prader-Willi syndrome using various techniques. Scoliosis.

[34] Odent T, Accadbled F, Koureas G, Cournot M, Moine A, Diene G (2008). Scoliosis in patients with Prader-Willi Syndrome. Pediatrics.

[35] Babady NE, Carelle N, Wells RD, Rouault TA, Hirano M, Lynch DR (2007). Advancements in the pathophysiology of Friedreich’s Ataxia and new prospects for treatments. Mol Genet Metab.

[36] Milbrandt TA, Kunes JR, Karol LA (2008). Friedreich’s ataxia and scoliosis: the experience at two institutions. J Pediatr Orthop.

[37] Reames DL, Smith JS, Fu KM, Polly DW, Ames CP, Berven SH (2011). Complications in the surgical treatment of 19,360 cases of pediatric scoliosis: a review of the Scoliosis Research Society Morbidity and Mortality database. Spine (Phila Pa 1976).

[38] Ialenti MN, Lonner BS, Verma K, Dean L, Valdevit A, Errico T (2013). Predicting operative blood loss during spinal fusion for adolescent idiopathic scoliosis. J Pediatr Orthop.

[39] McPhail GL, Howells SA, Boesch RP, Wood RE, Ednick M, Chini BA (2013). Obstructive lung disease is common in children with syndromic and congenital scoliosis: a preliminary study. J Pediatr Orthop.

